# Colorimetric Analysis of Transmitted Light Through Plasmonic Paper for Next-Generation Point-of-Care (PoC) Devices

**DOI:** 10.3390/bios15030144

**Published:** 2025-02-24

**Authors:** Caterina Serafinelli, Alessandro Fantoni, Elisabete C. B. A. Alegria, Manuela Vieira

**Affiliations:** 1 Lisbon School of Engineering (ISEL), Polytechnic University of Lisbon (IPL), Rua Conselheiro Emídio Navarro, nº1, 1959-007 Lisbon, Portugal; c.serafinelli@campus.fct.unl.pt (C.S.); afantoni@deetc.isel.ipl.pt (A.F.); elisabete.alegria@isel.pt (E.C.B.A.A.); 2 Centro de Química Estrutural, Institute of Molecular Sciences, Universidade de Lisboa (IST), 1049-001 Lisboa, Portugal; 3 UNINOVA-CTS and LASI, Quinta da Torre, Monte da Caparica, 2829-516 Caparica, Portugal; 4 NOVA School of Science and Technology, Quinta da Torre, Monte da Caparica, 2829-516 Caparica, Portugal

**Keywords:** POC devices, plasmonic paper, filtering properties, color spaces, light source

## Abstract

This study identifies the optimal conditions for enhancing the performance of the *Color Picker System*, a device designed for colorimetric sensing using plasmonic paper. A simulation study was conducted toanalyze the transmittance spectra of plasmonic paper embedded in different mixtures, resulting in a comprehensive color chart that includes the chromatic response as well as the RGB values of transmitted light. The filtering properties of the plasmonic paper were evaluated through colorimetric analysis, combining the transmittance characteristics with the spectrum of different light sources. Optimizing the correlation between these filtering properties and the light source enhances both sensitivity and precision. Arrays of nanoparticles with high absorbance, combined with Cold LED light sources, emerge as ideal components for the device set-up. Among the light sources tested, the White LED uniquely generates a red signal while producing the most significant variations in the green channel. In contrast, the Cold LED and Xenon Arc lamp produce the strongest colorimetric signals in the blue channel. This study provides a deep understanding of the filtering properties of plasmonic paper, opening a new way for the implementation of nanoparticle arrays in colorimetric sensing.

## 1. Introduction

The keystone of future precision medicine lies in the specific classification of patients based on their prognosis, predicted disease risk, and response to individualized therapy. This approach enables accurate and early diagnosis, increasing the opportunity for successful treatment and improving survival rates. Undoubtedly, the foundation of innovative medicine is the detection of validated biomarkers, and taking this into account, over the last decade, remarkable efforts have been directed toward the development of new biomarker detection technologies [1].

At the same time, the creation of next-generation sensing devices and their integration into routine clinical diagnosis has attracted growing interest as support for advanced medicine [2,3,4]. Point-of-care (PoC) devices, in particular, have experienced outstanding progress due to their inherent advantages. These devices require minimal sample quantities, often with little or no preparation, providing results in real time or faster results compared to common lab tests. Moreover, PoC devices can be used without specialized training or equipment, making them accessible and practical [5]. Consequently, research activities have been focused on simplifying analysis to create user-friendly devices suitable for use in home environments, beyond hospital and clinical settings.

A promising approach is colorimetric analysis, which exploits color changes as the analytical signal, often visible to the naked eye, without requiring the use of complex instrumentation or specialized personnel [6,7].

Nanomaterials have been pivotal in the development of innovative sensing devices, especially when exploiting interactions with light. Among these, noble metal nanoparticles stand out due to their remarkable optical properties, driving a revolution in nanosensing. The fascinating optical properties of metal nanoparticles are associated with their localized surface plasmon resonances (LSPRs). When particles are smaller than the wavelength of incident light, the interaction with the light’s electromagnetic field displaces conduction electrons from their equilibrium relative to the core ion and creates a dipole oscillating in resonance with the incident light wave [8]. As a result of these coherent collective electron oscillations, intense electromagnetic fields are confined to the particle surface, leading to strong light absorption at the LSPR wavelengths [9,10]. The position and bandwidth of the LSPR are influenced by factors such as nanoparticle size, morphology, and spacing, as well as the refractive index of the surrounding medium [11,12].

In sensing applications, the technology’s sensitivity to variations in the refractive index has proven to be a powerful tool to detect biorecognition events occurring on the particle surfaces, creating a new class of sensor, the LSPR-based biosensors [13,14]. A typical analysis relying on an LSPR sensor involves the spectral interrogation of an array of plasmonic nanoparticles, and the information is obtained by observing the changes in the response resulting from the interrogation with the analyte molecules.

Over the past two decades, plasmonic color technology has seen significant growth, finding implementation across a wide range of fields [15]. The use of metallic nanostructures to generate plasmonic colors has generated a remarkable increase in innovative colorimetric approaches for the effective detection of analytes [16,17].

Despite the effectiveness of LSPR analysis, given its good sensitivity and specificity, its full implementation in PoC devices is still a challenge because it requires bulky instrumentation and specialized personnel [18]. Stimulated by the increasing demand for a “home-made” approach for a fast and reliable analysis, the authors, in a previous work, developed a sensing device for the colorimetric detection of molecules based on plasmonic metal nanoparticles, referred to as the *Color Picker System* [19].

**Figure 1:**
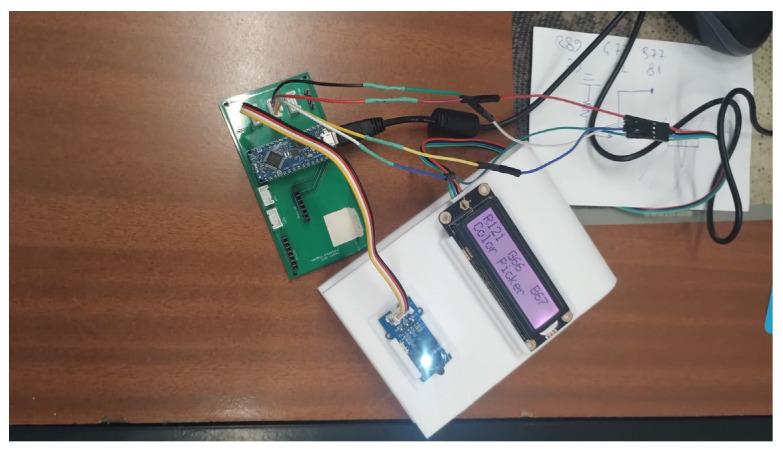
Picture of the *Color Picker System* set-up with the TCS34725 sensor, the RGB backlight DFRobot display, and the Arduino Nano microcontroller assembled onto a board. From Ref. [19].

The *Color Picker System* is a user-friendly device that is able to reproduce the color detected by the Grove I2C TCS34725 color sensor (SeedStudio, Shenzhen, China) in the backlight of an Grove LCD RGB backlight Display (SeedStudio, Shenzhen, China). Its objective is the colorimetric quantification of the analyte of interest, which permits sample analysis without the need for bulky instruments. The set-up of the Color Picker Systems is depicted in Figure 1. It includes the TCS34725 color sensor, an I2C RGB display, and an Arduino Nano microcontroller assembled on a board.

An essential component is the sensing platform, where color changes occur due to arrays of plasmonic nanoparticles. The nanoparticles generate the chromatic variations detected by the device due to their striking optical properties. The sensing mechanism is based on the measurement of the color change arising from the biorecognition event that occurs on the particle surface.

On this basis, the scaling up from colloidal particles to their integration into solid substrates facilitates their integration into the device. Concerning the nature of the substrate incorporating the metal nanoparticles, the last 20 years have gone through an evolution, starting at the exclusive utilization of glossy substrates, passing across rigid polymers, and arriving at flexible substrates such as polymeric films, hydrogels, and cellulosic materials [20]. Also, it is noteworthy that hybrid materials composed of plasmonic nanoparticles dispersed into polymeric matrixes, such as gels, hydrogels, and solid polymers, are attracting increasing attention, thus accelerating their development as sensing devices [21].

Among the various materials, cellulosic substrates have emerged as particularly promising candidates for point-of-care (PoC) sensing devices due to their natural abundance, low cost, and unique properties [22,23]. In addition, due to their intrinsic capillarity, the utilization of additional supports and tools such as microfluidic channels, pumps systems, reaction chambers, etc., are not required. The inherent white/pale color of cellulose intensifies any variation in color, thus facilitating a colorimetric evaluation and analysis. Given these advantages, plasmonic paper–cellulose integrated with gold nanoparticles (AuNPs) embedded within its fiber network has been adopted as the sensing in the *Color Picker System.* By detecting color differences in the plasmonic paper, the device can identify the presence of target analytes. The device is in its early stage, but it has been tested to detect the chromatic changes in the plasmonic paper that appear as a result of the different refractive index (RI) around the particle surface [19]. AuNPs have been used as a proof of concept due to their chemical stability, biocompatibility, and strong plasmonic resonance in the red to near-infrared (NIR) range, which is ideal for biomedical applications. In future studies, other metal nanoparticles, namely, AgNPs, with strong plasmonic responses in the visible range and high sensitivity for colorimetric detection, should also be tested.

**Figure 2:**
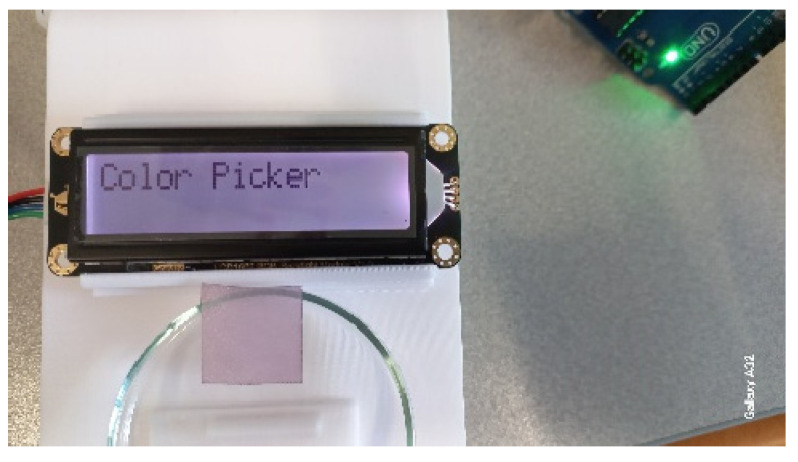
The *Color Picker System*, reproducing in the backlight of the I2C, displays the color of plasmonic paper wetted in glycerol. From Ref. [19].

Figure 2 shows that the Color Picker System can reproduce the color of plasmonic paper wetted in glycerol.

While previous studies [24] have investigated plasmonic paper for quantitative analysis based on the colorimetric detection of molecules, to the best of our knowledge, no evaluations of particle properties to enhance the method’s performance have been performed. This gap in the literature underscores the need for further exploration. At the same time, the TCS34725 color sensor was evaluated for the quantitative analysis of colored solutions [25,26], and it was found that the sensor achieved analytical performance similar to that obtained with the commercial spectrophotometer (Table 1).

Aiming to take the device one step forward for full implementation in real-world applications, herein, we optimize the correlation between the filtering properties of the plasmonic paper and the spectrum of the source light to increase the sensitivity and precision of the Color Picker System to achieve analytical performances similar to spectroscopic approaches. However, the present study also provides a better understanding of the plasmonic particle properties affecting their chromatic response, thus creating a tool to improve the implementation of devices for colorimetric sensing based on arrays of plasmonic nanoparticles integrated into a solid support, such as paper.

## 2. Materials and Methods

### 2.1. Materials

#### 2.1.1. Plasmonic Paper

Tetrachloroauric acid (HAuCl_4_·3H_2_O), tri-sodium citrate, and glycerol were purchased from Sigma-Aldrich (Darmstadt, Germany) and used as received. Common filter paper, typically used in chemical laboratories, was used for the preparation of plasmonic paper.

#### 2.1.2. Light Sources

Commercial Cold LED, White LED, and multi-LED were used as light sources. The spectrum of the SLS603 Xenon Arc lamp, MBB1L3 Broadband LED, and QTH10 Quartz Tungsten Halogen Lamp, all from Thorlabs, were downloaded from the producer website.

#### 2.1.3. Optical Spectra

Power intensity spectra were recorded with a Thorlabs CCS200 Spectrometer (Newton, NJ, USA).

#### 2.1.4. Characterization of AuNPs by TEM

The AuNPs were characterized by transmission electron microscopy (TEM), and images were acquired with a JEOL 1200EX (Tokio, Japan).

#### 2.1.5. Characterization of Plasmonic Paper

Transmittance spectra were measured using a Shimadzu UV-2501PC (Kyoto, Japan) spectrometer, whereas scanning electron microscopy (SEM) images were acquired with a Phenom ProX G6 from Thermofisher (Waltham, MA, USA).

### 2.2. Methods

#### 2.2.1. Synthesis of Citrate-Capped AuNPs

The dispersion of citrate-capped AuNPs was prepared by adding 0.5 mL of a 1% stock solution of hydrogen tetrachloroaurate (III) trihydrate to 50 mL of water. The solution was equipped with a refrigerator and heated under stirring until boiling. Then, 0.423 mL of a 34 mM solution of trisodium citrate dihydrate was rapidly added to the flask; after boiling for 5 min, the solution was cooled down. The appearance of a reddish color indicated the successful formation of nanoparticles.

#### 2.2.2. Plasmonic Paper Substrate Preparation

The plasmonic paper was prepared by grafting the gold nanoparticles (AuNPs) onto a piece of filter paper, as previously reported [27]. Briefly, a strip of common laboratory filter paper (7 × 13 mm) was immersed for 24 h in a dispersion of AuNPs and left overnight at 4 °C for about 24 h. The paper strip was removed from the solution, washed with MilliQ water, and dried for about 2 h in an oven at about 30 °C.

#### 2.2.3. Dependence of Transmittance Spectra on Refractive Index Measurements

The refractive index of the dielectric environment surrounding the AuNPs was changed using ethanol–glycerol mixtures with different glycerol concentrations. The refractive index of these mixtures was determined using the Lorentz–Lorentz formula (Equation (1)) [28]:

(1) $$\frac{n_{12}^{2}-1}{n_{12}^{2}+2}=\varphi_{1}\frac{n_{1}^{2}-1}{n_{1}^{2}+2}+\varphi_{2}\frac{n_{2}^{2}-1}{n_{2}^{2}+2}$$

where $n_{1}$ and $n_{2}$ are the refractive indices of ethanol (1.36) and glycerol (1.47), $\varphi_{1}$ and $\varphi_{2}$ are their volume fractions, and $n_{12}$ is the refractive index of the mixture. The compositions of the mixtures, along with their refractive indices, are reported in Table 2:

The plasmonic paper was wetted with each of the mixtures, and the corresponding transmittance spectra were measured.

#### 2.2.4. Simulation Study

The overall simulation study was performed by means of a MATLAB (version 2024) script (Mathworks) developed by us for this purpose.

In the first phase, we combined the measured transmittance spectra of the different plasmonic papers with the normalized power intensity spectra of the light source to calculate the spectra of the transmitted light. Also, the chromatic response was represented along with the spectra of the transmitted light.

In the subsequent phase, the spectra of the transmitted light were converted into vectors in the xy, L*a*b*, and RGB color spaces: the main output was the representation of the transmitted light, along with the light source as reference, in the chromaticity diagram.

**Figure 3:**
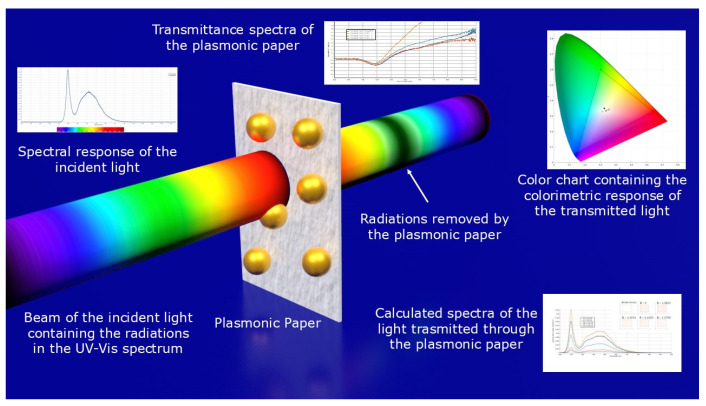
Schematics representing the principal components of the simulation study performed on the light transmitted through the plasmonic paper.

In Figure 3, the plasmonic paper is depicted along with the inputs (spectrum of the incident light and transmittance spectra of the plasmonic paper) and the outputs (chromaticity diagram with the chromatic response of the incident and transmitted light and the calculated spectra of the transmitted light along with their color representations). The beam of the incident light is represented with the full UV-Vis spectrum, whereas the dark band in the transmitted light represents the radiation removed by the plasmonic paper.

##### CIE 1931 RGB Color Space and CIE 1931 XYZ Color Space

The CIE 1931 RGB and CIE 1931 XYZ color spaces were developed by the International Commission on Illumination (CIE) in 1931, based on experiments conducted in the late 1920s by William David Wright and John Guild [29]. The results were combined into the specifications of the CIE RGB color space, from which the CIE XYZ color space was derived [30].

Whereas R, G, and B are real light sources with known specifications, X, Y, and Z are theoretical sources that are more saturated compared to any real light source and allow for the matching of any real color utilizing a positive amount of the three primaries [31].

##### From Spectral Response to XYZ Color Space: CIE 1931 Matching Functions

The formulation at the basis of the simulation study relies on the CIE color matching functions $\bar{x}\left(\lambda \right),\;\bar{y}\left(\lambda \right),\;\mathrm{and}\;\bar{z}\left(\lambda \right)$. The color matching functions in the CIE 1931 XYZ Color space have non-negative values [32], as expected, and were determined taking into account the color perceived from a sample of observers over the visual range from λ_violet_ = 300 to λ_red_ = 780 nm. They can be considered weight factors when calculating the CIE XYZ tristimulus values. Given a spectral color distribution, I(λ), the CIE XYZ values are calculated by summing, over the visual range, the product of the color matching function weights at each wavelength and the intensity emitted at a constant narrow wavelength interval centered there (Equations (2)–(4)) [33,34].

(2) $$X=\int_{300}^{780}I\left(\lambda \right)\bar{x}\left(\lambda \right)d\lambda$$

(3) $$Y=\int_{300}^{780}I\left(\lambda \right)\bar{y}\left(\lambda \right)d\lambda$$

(4) $$Z=\int_{300}^{780}I\left(\lambda \right)\bar{z}\left(\lambda \right)d\lambda$$

##### Chromaticity Diagram

Another way to present the tristimulus data is to calculate the proportion of each value relative to the sum of all three (Equations (5)–(7)) [35]:

(5) $$x=\frac{X}{\left(X+Y+Z\right)}$$

(6) $$y=\frac{Y}{\left(X+Y+Z\right)}$$

(7) $$z=\frac{Z}{\left(X+Y+Z\right)}$$

But it is possible to further simplify the xyz representation considering the relation (Equation (8)) [36]:

(8) x+y+z=1

Considering Equation (8), only two coordinates, usually *x* and *y*, are required to describe the chromatic response, or chromaticity, of light. This simplifies the color representation from three dimensions to two. Nevertheless, in the procedure, the information on the absolute luminance *Y* is lost, and the derived color space specified by *x*, *y*, and *Y* is referred to as the CIE xy color space, which is widely used in practical applications.

**Figure 4:**
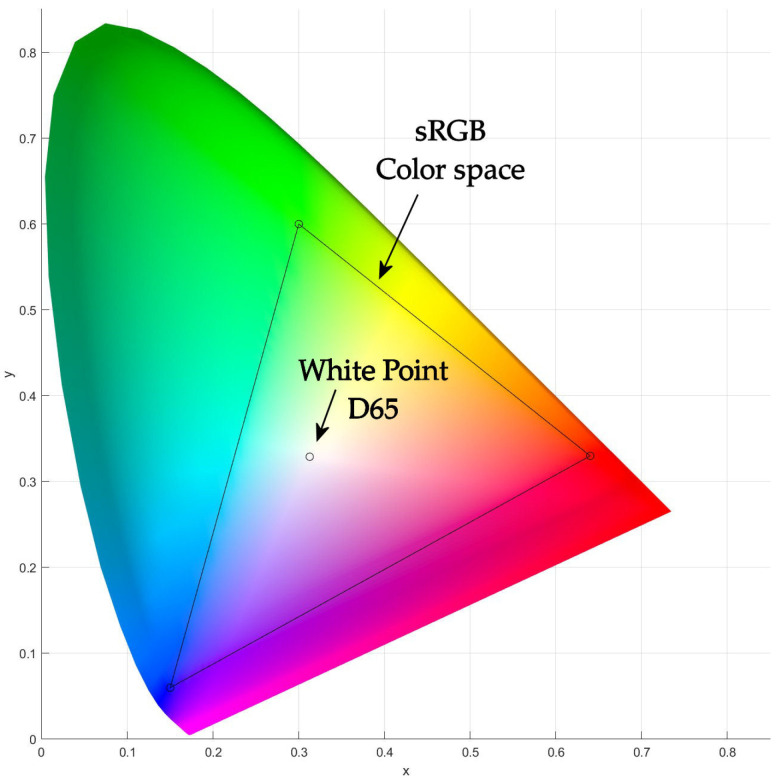
Representation of the CIE 1931 chromaticity diagram along with the sRGB color space and white point.

When plotting the pure monochromatic colors of the spectrum in their xy notation, a horseshoe diagram is obtained, referred to as the CIE 1931 color space chromaticity diagram (Figure 4). The outer curved boundary is the spectral (or monochromatic) locus and represents monochromatic light: each point corresponds to the pure hue of a single wavelength. The straight line at the base of the horseshoe, known as the “line of purples”, represents mixtures of red and blue light [37,38]. All other non-monochromatic or less saturated colors are located inside the horseshoe, representing all chromaticities perceptible to the average person. This region is called the human vision gamut [39]. These are displayed in color, and a very interesting property of the CIE chromaticity diagram is that it can be employed to identify the color resulting from the mixing of two known emissive colors: adjusting the ratio between the two lights will make the resulting color move along the line [40]. This concept can be extended to three light sources.

The light sources are defined as primary when it is not feasible to simulate one of them by mixing the two others, which usually are narrow band light sources [41]. Once selected, based on their requirements, the primaries enclose a triangular shape in the CIE chromaticity diagram [42]. When mixing the primaries in variable proportions, all the colors inside the triangle are generated, also called the color gamut. Herein, the triangle is shown inside the color chart since it represents the colors detected by the TCS34725 color sensor.

##### From CIE 1931 XYZ Color Space to RGB Color Space

A further conversion from CIE 1931 tristimulus to RGB values requires a transformation by means of the appropriate chromaticity matrix. From a geometric point of view, the chromaticity matrix maps the points inside the chromaticity diagram onto the subset of points within the RGB gamut, i.e., the indicated triangular region. Once defined along with the vertices of the triangle determining the RGB color system (the chromaticity of the three primary colors), as well as the white point, the CIE XYZ tristimulus values can be gathered into a matrix (Equation (9)) [43,44].

(9) $$T=\left(\begin{array}{ccc} X_{R} & X_{G} & X_{B}\\ Y_{R} & Y_{G} & Y_{B}\\ Z_{R} & Z_{G} & Z_{B} \end{array}\right)$$

Supporting the conversion between the RGB color space and tristimulus values by means of Equation (10):

(10) $$\left(\begin{array}{c} X\\ Y\\ Z \end{array}\right)=T\left(\begin{array}{c} R\\ G\\ B \end{array}\right)$$

along with the inverse relationship (Equation (11)):

(11) $$\left(\begin{array}{c} R\\ G\\ B \end{array}\right)=T^{-1}\left(\begin{array}{c} X\\ Y\\ Z \end{array}\right)$$

the RGB values are calculated by multiplying the inverse matrix $T^{-1}$ by the tristimulus values.

##### sRGB Color Space

Herein, the CIE chromaticity diagram is shown alongside the sRGB color space. The standard RGB color space (sRGB) is the most widespread color system, which was developed in 1996 through the cooperation activities of Microsoft Corporation and Hewlett-Packard. It is a straightforward response to the increasing need for standardization of color in illuminated screens and computers [45,46].

The color gamut that can be represented in the sRGB color space is the color triangle defined by the ITU-R BT.709 primaries [47]. The white point of the sRGB color space is reliant on the standard CIE D65 illuminant [48], which specifies natural daylight with an overcast sky or a mix of lights arising when direct sunlight and light from the remaining sky fall on a horizontal surface. At the same time, the standard CIE D65 illuminant corresponds to a black-body radiator at a correlated color temperature (CCT) of 6500 K [49,50]. Table 3 displays the coordinate in the xy color space for the ITU-R BT.709 primaries and D65 illuminant [51,52]. In Figure 4, the triangle representing the sRGB color space is represented along with the white point into the chromaticity diagram (CIE standard D65).

##### Color Difference

The color difference in this study was calculated using the CIE76 standard. To standardize color difference measurements, the International Commission on Illumination (CIE) recommends the use of two color spaces along with their associated color difference formulas: the CIE 1976 (L*a*b*) and CIE 1976 (L*u*v*) formulas [53,54].

The CIE 1976 Lab color space is defined in terms of the L, a, and b tristimulus values, where L represents the lightness and ranges from 0 to 100 (from black to white) and a and b are the chromaticity components: a represents the redness and greenness ranging from −128 to +127 (from negative green to positive red) and b represents the yellowness and blueness ranging from −128 to +127 (from negative blue to positive yellow) [55].

Also, the RGB and CIE 1931 XYZ color spaces are not perceptually uniform, so they are not able to reproduce all the observed color [56], whereas the CIE 1976 Lab color space presents an improved uniformity, which is a more suitable color space for accurate color difference identification [57,58]. Considering two colors in the CIELAB color space, $L_{1}^{*}a_{1}^{*}b_{1}^{*}$ and $\ddot{L_{2}^{*}}a_{2}^{*}b_{2}^{*},$ the CIE76 color difference, $∆E_{L^{*}a^{*}b^{*}},$ is calculated by the formula (Equation (12)) [59,60]:

(12) $$\Delta E_{L^{*}a^{*}b^{*}}=\sqrt{{\left(\Delta L^{*}\right)}^{2}+{\left(\Delta a^{*}\right)}^{2}+{\left(\Delta b^{*}\right)}^{2}}$$

according to the Euclidian distance among two points in the CIE-L*a*b* color space.

## 3. Results and Discussion

### 3.1. Plasmonic Paper Characterization and Effects of Local Dielectric Environment

**Figure 5:**
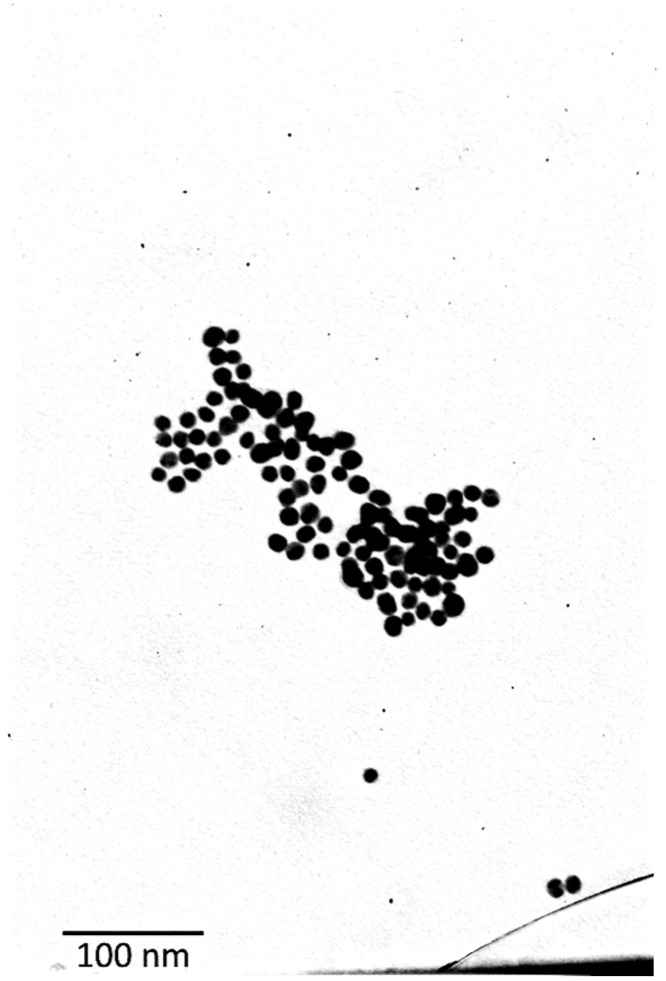
TEM image of the citrate-capped AuNPs.

The synthesized AuNPs were first characterized using transmission electron microscopy (TEM). Figure 5 presents a TEM image of the gold nanoparticles, revealing key morphological aspects. Most of the nanoparticles exhibit a semi-spherical to oval shape, which is characteristic of nanoparticles. Additionally, the image indicates a relatively narrow size distribution (10–20 nm), as most nanoparticles appear to have similar dimensions.

**Figure 6:**
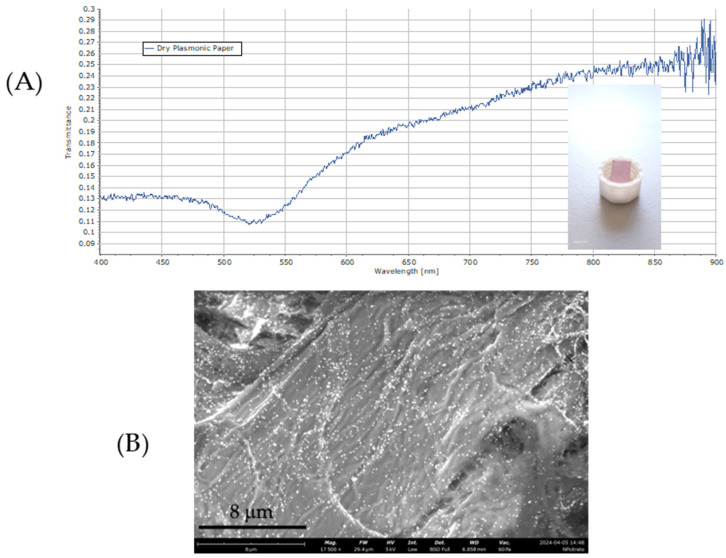
Transmittance spectrum (**A**) and the SEM image (**B**) of the dry plasmonic paper.

The optical properties of the plasmonic paper were initially characterized using transmittance spectroscopy, while its morphological features were examined via scanning electron microscopy (SEM). The transmittance spectrum depicted in Figure 6A exhibits a prominent dip centered at 525 nm, indicative of localized surface plasmon resonance. The SEM image in Figure 6B shows a uniform distribution of gold nanoparticles within the three-dimensional network of the paper’s cellulosic fibers, with no visible signs of nanoparticle aggregation.

**Figure 7:**
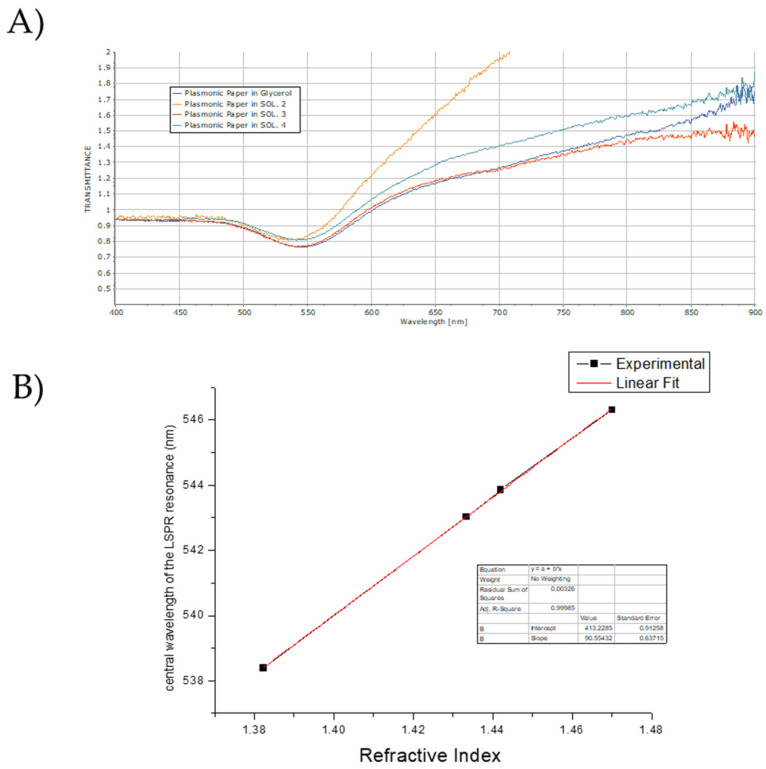
(**A**) Transmittance spectra of plasmonic papers embedded in solutions with different RIs; (**B**) graph reporting the position of the minimum transmittance wavelength against the RI.

To assess the impact of changes in the dielectric environment on the localized surface plasmon resonance (LSPR) of the plasmonic paper, we first used a spectroscopic approach. The refractive index of the medium surrounding the particle surface was progressively changed by using ethanol–glycerol mixtures with different ethanol percentages. For each solution, the plasmonic paper was wetted, and the transmittance spectra were measured and shown in Figure 7A. All spectra were normalized at 400 nm, where absorbance is mainly due to inter-band transitions, thus facilitating comparison.

By plotting the position of the transmittance dip versus the RI of the medium embedding the plasmonic paper, it is possible to observe a red shift in the transmittance, in accordance with the Mie theory (Figure 7B). Also, the graph shows that a reasonably good linear fit could have been reached for the dependence of the transmittance position from the calculated RI values.

### 3.2. Spectral Response of the Source Light and the Transmitted Light

**Figure 8:**
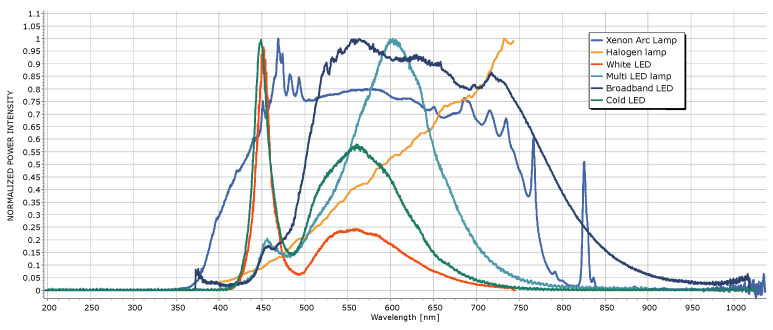
Normalized power intensity spectra for the different light sources.

The illumination with lamps originating different power spectra produces different chromatic responses in the same sample. In light of that, we considered different light sources to simulate the effects of the incident light on the color recognition process. Also, in order to evaluate the sensing properties of the method, the effect of the illuminant was evaluated in combination with the effect of the dielectric environment, calculating the spectra of the transmitted light at different RIs for each type of light source. In Figure 8, the normalized power intensity spectra of the different light sources before crossing the plasmonic papers embedded in the different mixtures are reported.

In the spectra of the LED lamps (White, Multi, Broadband, and Cold), two distinct peaks are noticeable. One peak is centered around 450 nm, while the other, broader peak occurs at longer wavelengths.

Currently, most LEDs use phosphor conversion technology (phosphor-converted LEDs or pcLED) [61]. White light is generated by combining a portion of the blue light emitted directly from an LED with a Gallium Nitride (GaN) substrate (smaller peak at 451 nm) with the light converted to yellow emitted by the phosphors (broad peak at 567 nm). These phosphors can be located on the LED’s emitting surface, within the encapsulant, or away from the LED (remote phosphors). In the case of the Cold and White LEDs, the peak centered at smaller wavelengths presents the highest value for the intensity, whereas the intensity of that peak is notably reduced in the case of the Broadband and Multi-LED lamps. The peak centered at larger wavelengths in the spectra of the Broadband LED is wider compared to the peaks of the other LED lamps. The spectrum of the Halogen lamp is characterized by the absence of peaks, while the power intensity grows for increasing wavelengths. In contrast, the Xenon Arc lamp exhibits a broadband spectrum.

**Figure 9:**
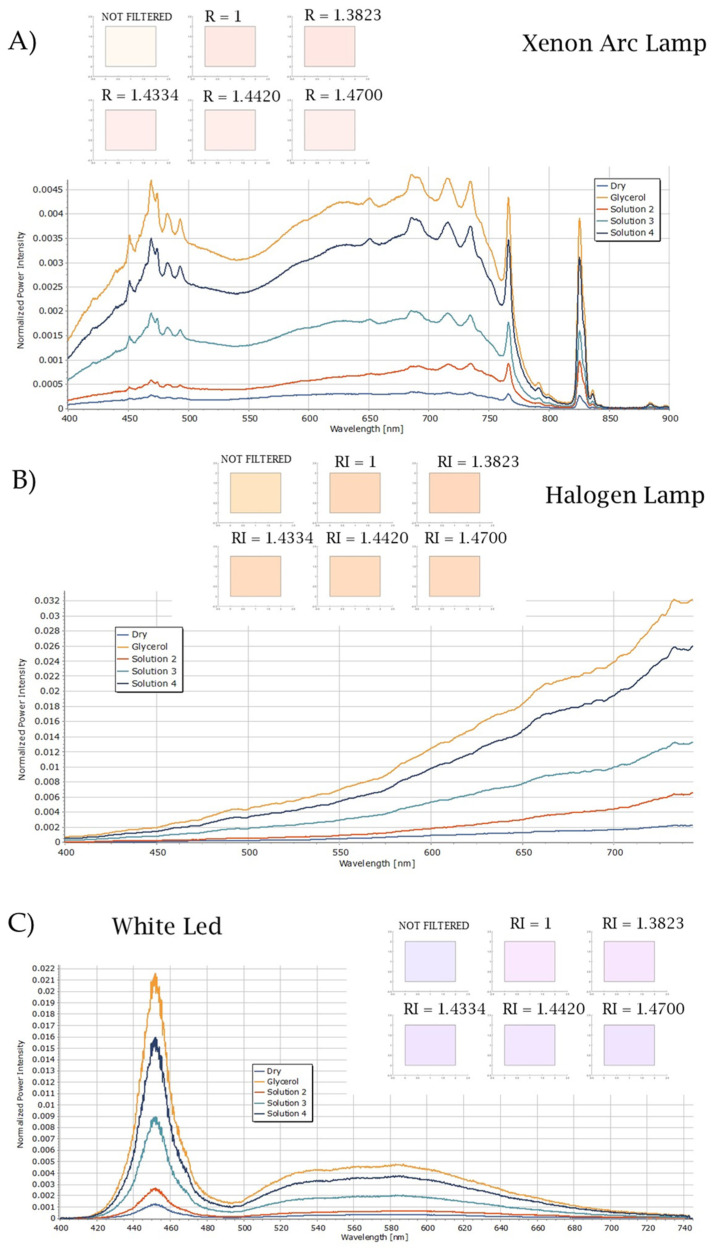

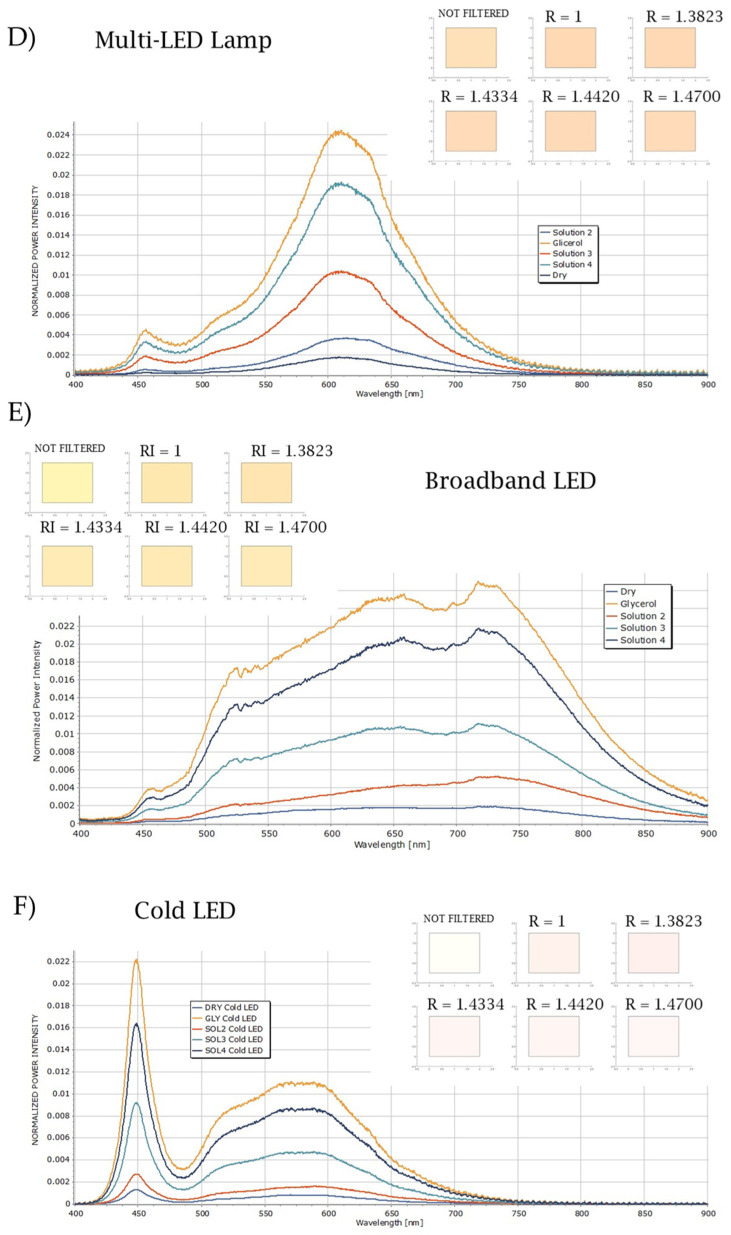
Calculated spectra of light filtered by the different plasmonic papers, as well as their chromatic responses, when using the (**A**) Xenon Arc lamp, (**B**) Halogen lamp, (**C**) White LED lamp (**D**) Multi-LED lamp, (**E**) Broadband LED lamp, and (**F**) Cold LED lamp as light source. The chromatic response of the filtered light is shown.

Figure 9 displays the calculated spectra for light transmitted through the plasmonic paper embedded in different media. The RI values of the different mixtures used to wet the plasmonic paper are reported, where R = 1 is the RI of the air, indicating the dry plasmonic paper.

For each type of light source, it can be observed that, according to the higher values for the RI of the medium embedding the plasmonic paper, the power of the transmitted light spectra increases. Because the calculated spectra of the transmitted light result from a combination of the light source spectra with the transmittance spectra of the plasmonic paper, they display the characteristics of both types of spectra. This is particularly evident when using the Xenon Arc lamp as the light source (Figure 9A); in that case, it is possible to observe the dip in the plasmonic paper transmittance in the calculated spectra of the transmitted light, which is more pronounced and thus more representative of the transmittance of the plasmonic paper, when the values of RI increase.

For the other illuminants, the dip in the transmittance is not evident in the spectra of the transmitted light. Along with the spectra in the inset of Figure 9, the chromatic response of the light source and the transmitted light are reported to illustrate the difference in the colors when changing the dielectric environment around the particle surface.

### 3.3. Filtering Properties of the Plasmonic Paper

The metal nanoparticles integrated into the three-dimensional structure of the plasmonic paper, which absorb the electromagnetic radiations in the range of the LSPR wavelengths, act as a stop-band filter by removing from the incident light the absorbed radiations, with the final filtering properties arising from the combination between the wavelengths of the minimum and intensity of transmittance.

The graph in Figure 7A,B shows that the wavelength of the absorbed radiation increases with the RI values according to the following order of the medium embedding the plasmonic paper: glycerol > solution 4 > solution 3 > solution 2 > dry. Here, the plasmonic paper embedded in glycerol presents the larger wavelengths of absorption, whereas the dry plasmonic paper presents the smallest.

Regarding the intensity, the dry plasmonic paper has the lowest value for the transmittance, followed by the plasmonic papers embedded in solution 2 and in solution 4, whereas the transmittance of the plasmonic paper wetted in solution 3 and glycerol are the highest and are comparable. The transmittance properties of the plasmonic paper are summarized in Table 4, where the wavelengths and the intensity of the transmittance are reported in decreasing order.

From the data reported in Table 4, it turns out that, according to the highest value of the intensity and the minimum wavelengths of the transmittance, the plasmonic paper embedded in glycerol removes the lowest portion of radiation with the longest wavelengths from the incident light, whereas, based on the lowest values, the dry plasmonic paper removes the largest part of radiations with shortest wavelengths.

The transmittance intensity of the plasmonic paper embedded in glycerol is comparable to that of the plasmonic paper embedded in solution 3, but the minimum is centered at lower wavelengths. This implies that the plasmonic paper embedded in glycerol and the plasmonic paper embedded in solution 3 remove the same quantity of radiations from the incident light but with different wavelengths. Therefore, the differences in the transmitted light arise mainly from the wavelength of the adsorbed radiations. In a similar way, the value of the transmittance intensity of the plasmonic paper embedded in solution 2 is comparable to that of the plasmonic paper embedded in solution 4; however, the peak is centered at shorter wavelengths, thus originating the variations in the transmitted light.

### 3.4. Shift Toward the Red, Green, and Blue Zones of the Color Chart

**Figure 10:**
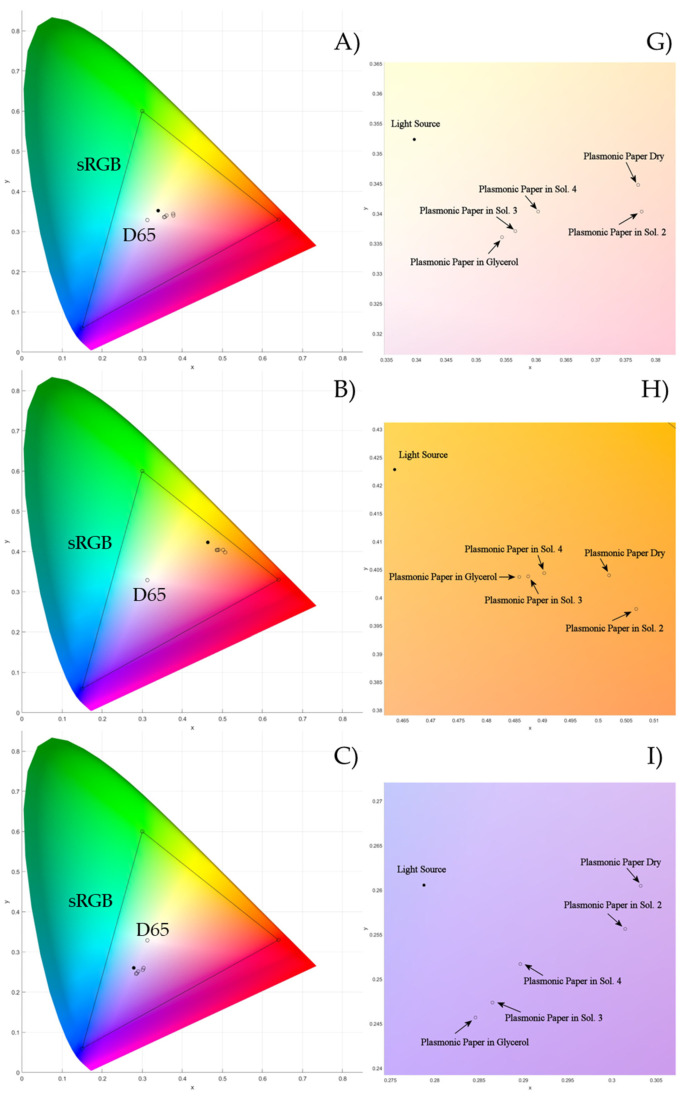

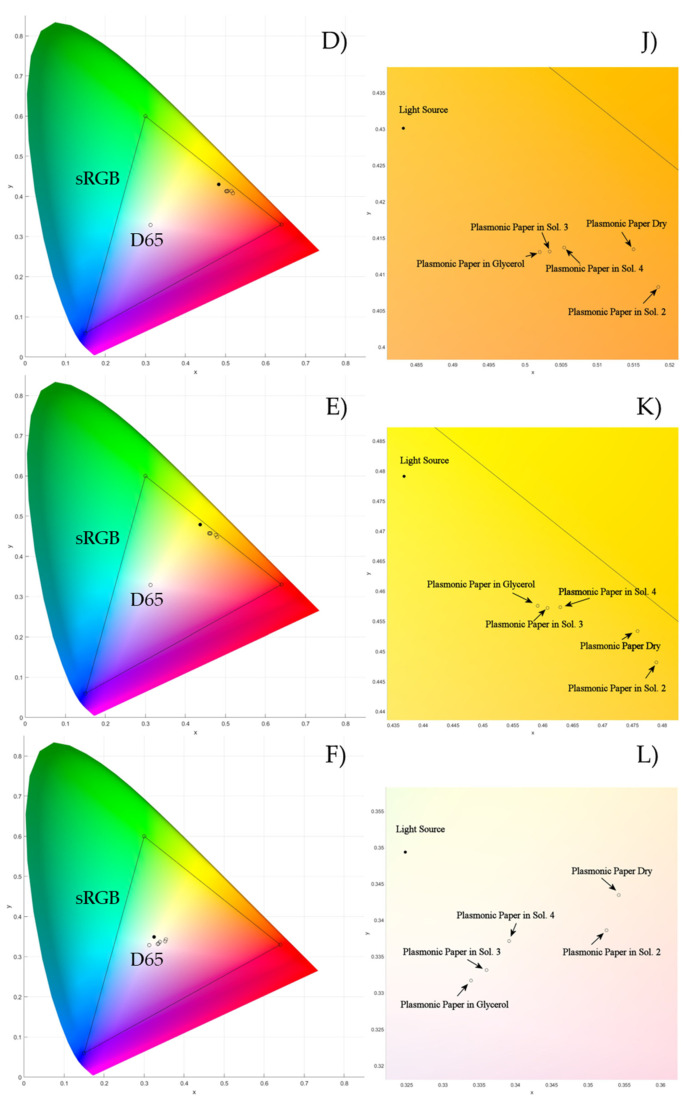
Color representation of the light transmitted through the plasmonic papers embedded in different dielectric media (left side) as well as the focus on the area of the color chart containing the light source and the transmitted light (right side): Xenon Arc lamp (**A**,**G**), Halogen lamp (**B**,**H**), White LED (**C**,**I**), Multi-LED (**D**,**J**), Broadband LED (**E**,**K**), and Cold LED (**F**,**L**).

The position in the *xy* color space of the transmitted light through the plasmonic paper embedded in the different mixtures, along with the light source (black circle) as a reference, are reported in Figure 10 (left side), as well as the details of their localization (right side). The main parameters affecting the properties of the transmitted light will be discussed in light of the way in which it moves closer to the red, green, and blue zones of the color chart, but also considering the way they approach the light source. It is possible to individuate two sets of data. When the White LED is used, a sequence different from that obtained with the other light sources is obtained, and the different data will be discussed separately.

#### 3.4.1. Shift for Illuminants Different from White LED

In Table 2, the sequences followed by the transmitted light to move closer to the red, green, and blue zones of the color chart are reported according to the different media embedding the plasmonic paper when using light sources different from the White LED.

The first term of the sequence is the point that is more distant from the considered zone of the color chart, whereas the last term is the closest.

Firstly, the way in which the transmitted light moves closer to the red zone of the color chart is considered. From Table 5, it is possible to observe that the light transmitted through the plasmonic paper embedded in glycerol is the most distant from the red zone of the color chart. According to the discussed filtering properties, the particles of the plasmonic paper embedded in glycerol remove the lowest portion of radiation with the longest wavelengths so that the distance of the transmitted light from the red zone is the greatest of the sequence.

The plasmonic paper embedded in solution 3 absorbs the same quantity of radiation as the plasmonic paper embedded in glycerol, though with shorter wavelengths; therefore, an increased quantity of radiation with larger wavelengths remains in the transmitted light, resulting in further advancement toward the red zone of the color chart. In the same way, the plasmonic paper embedded in solution 2 and the plasmonic paper embedded in solution 4 absorb radiations with shorter wavelengths from the incident light, but in larger quantities compared to that of the plasmonic paper wetted in glycerol. This results in a higher quantity of radiations with longer wavelengths remaining in the transmitted light.

Finally, it is possible to note that the dry plasmonic paper is not the last term of the sequence, as may be expected from the lowest values of the intensity and the minimum wavelengths of the transmittance. The value of the transmittance intensity of the plasmonic paper wetted with solution 2 is higher compared to that of the dry plasmonic paper, so the quantity of removed radiation prevalently affects the properties of the transmitted light. Likewise, it is possible to observe that the plasmonic paper embedded in solution 3, considering the higher intensity but lower values of the minimum compared to that of the plasmonic paper embedded in solution 4, has a stronger effect on determining the amount of radiation with longer wavelengths in the transmitted light.

In such circumstances, the sequence followed by the transmitted light to move closer to the red zone of the color chart relies mostly on the relative quantity of radiation with longer wavelengths remaining in the light after being filtered: their volume in the transmitted light is larger, and the distance from the red zone of the color chart is shorter.

It can be observed from Table 6 that the sequence in which the transmitted light approaches the red zone of the color chart is the reverse of the sequence in which it approaches the green zone. Figure 8 shows that, for light sources other than the White and Cold LEDs, a significant portion of the spectrum is concentrated above 600 nm, indicating that most of the incident light consists of radiation within the red region of the UV-Vis spectrum.

Due to the filtering properties of the plasmonic paper, the relative proportion of shorter wavelength radiation in the transmitted light changes. Since the green region of the UV-Vis spectrum corresponds to shorter wavelengths compared to the red region, an increase in the proportion of shorter wavelengths in the transmitted light results in a reduced shift toward the green zone of the color chart.

Whereas the advancement toward the red zone relies on the relative volume of radiation with longer wavelengths remaining in the transmitted light, the shift toward the green zone depends on the relative quantity of radiation with smaller wavelengths. Next, we discuss the advancement of the transmitted light toward the blue zone of the color chart. When the incident light crosses the plasmonic paper wetted with glycerol, the lowest portion of radiation with longest wavelengths is removed in such a way that the highest quantity of radiation with shorter wavelengths is present in the transmitted light. As a result of the increased volume of radiation with smaller wavelengths, the distance from the blue zone of the color chart is the shortest of the sequence. A comparable quantity of radiation is removed from the incident light by the plasmonic paper embedded in solution 3 but with shorter wavelengths; therefore, a lower portion of radiation with smaller wavelengths remains in the transmitted light, causing an increase in the distance from the blue zone of the color chart.

Similarly, when the incident light passes through the plasmonic papers wetted in solution 2 and in solution 4, a comparable amount of radiation is absorbed, but at a shorter wavelength than in glycerol. This results in a reduced fraction of shorter wavelength radiation remaining in the transmitted light. Furthermore, since the plasmonic paper soaked in solution 2 absorbs more short-wavelength radiation, an even smaller amount remains in the transmitted light, leading to a greater shift away from the blue zone of the color chart. Finally, the dry plasmonic paper absorbs the largest portion of the shortest wavelength radiation, leaving the smallest amount of short-wavelength radiation in the transmitted light.

Due to the reduced amount of short-wavelength radiation, the transmitted light is furthest from the blue zone of the color chart in this sequence. In this context, the proximity of the transmitted light to the blue zone of the color chart depends primarily on the amount of short-wavelength radiation it contains after filtering: the greater the quantity of these shorter wavelengths, the closer the light moves to the blue area of the color chart.

#### 3.4.2. Shift for the White LED

Despite the regularity displayed by the different illuminants, when using the White LED lamp as a light source, the order according to which the transmitted light approaches the red zone and green zone of the color chart is different from the other illuminants (Table 6).

As already discussed, a peak of high intensity centered at about 450 nm is present in the spectrum of the White LED, meaning that a greater part of the incident light contains radiation with wavelengths composed of the blue region of the UV-Vis spectrum, so that even the properties of the transmitted light are controlled by this kind of radiation. In this case, the effect of the plasmonic paper is to regulate the relative quantity of radiation with shorter or longer wavelengths in the transmitted light, thus determining the advancement toward the different areas of the color chart.

Considering the shift toward the red zone of the color chart, the plasmonic paper embedded in glycerol removes the smallest amount of long-wavelength radiation. As a result, the transmitted light contains a higher proportion of short-wavelength radiation compared to the incident light, which increases its relative quantity. This leads to the largest distance from the red zone in the sequence. Similarly, the dry plasmonic paper absorbs the greatest amount of radiation, leaving the transmitted light with the lowest proportion of short-wavelength radiation, resulting in a better proximity to the red zone of the color chart.

When observing the shift toward the blue zone, the distance decreases as the relative amount of short-wavelength radiation in the transmitted light increases, consistent with the behavior seen with the other illuminants. It is also evident that the sequence for approaching the green zone follows the same order as for the red zone.

Since the green region of the UV-Vis spectrum corresponds to longer wavelengths than the blue region, a decrease in short-wavelength radiation and an increase in long-wavelength radiation in the transmitted light result in a reduced proximity to the green zone on the color chart.

The shift toward the blue zone is determined by the amount of long-wavelength radiation removed from the incident light by the plasmonic paper. In contrast, the shift toward the red and green zones depends on the relative proportion of long- or short-wavelength radiation in the transmitted light, as well as the spectral distribution of the incident light. A higher proportion of short-wavelength radiation in the transmitted light causes a shift toward the shorter wavelength region of the UV-Vis spectrum compared to the incident light. Thus, considering the shifts toward the red, green, and blue zones of the color chart, the chromatic response of the transmitted light depends on the intensity and wavelength of the plasmonic paper’s minimum transmittance combined with the spectral response of the light source. This indicates that controlling both the transmittance properties of the plasmonic paper and the light source spectrum is crucial for optimizing the performance of the colorimetric device.

The effect of the plasmonic paper is to regulate the relative quantity of radiation with shorter or longer wavelengths in the transmitted light, thus determining the shifts toward the different areas of the color chart. It also implies that when the quantity of radiation removed from the system of plasmonic nanoparticles increases, the difference in the chromatic response of the transmitted light is larger. By transferring these results into the design of the Color Picker System, we conclude that systems of plasmonic particles with high absorbance are preferred when preparing the plasmonic paper. They will produce a larger change in the color of the transmitted light, enhancing the sensitivity of the device. Furthermore, the results also suggest that to achieve greater signal reproducibility, it is important to measure the transmittance spectrum at the same location on the plasmonic paper. Different regions may contain varying numbers of AuNPs, leading to differences in transmittance intensity that can affect the chromatic response and the accuracy of the measurements. In terms of the light source spectrum, a more detailed evaluation was performed based on the distances between two consecutive points representing the transmitted light on the color chart.

### 3.5. Shift Toward the Light Source

At first sight, it is possible to note from Figure 10 that the filtered light is localized at a shorter distance from the red zone of the color chart with respect to the incident light. This trend holds for all the considered light sources and could be related to the wavelength of the radiation removed by the plasmonic paper that is localized in the green region of the UV-Vis spectrum. Due to a lower relative quantity of that type of radiation, the filtered light moves closer to the proximity of the red zone of the color chart. However, it is possible to observe that the order followed by the transmitted light to shift toward the light source when using the White and Cold LEDs is different from the other.

#### 3.5.1. Approach for Illuminants Different from the White LED and Cold LED Lamps

In Table 7, the order according to which the transmitted light moves closer to the light sources different from the White and Cold LEDs is reported.

Comparing the data in Table 7 with those in Table 5, it becomes evident that the distance from the light source decreases following the same order as the sequence followed to approach the green zone of the color chart. This suggests that the sequence by which the filtered light approaches the light source in the color chart does not depend on the amount of radiation removed from the incident light. If that were the case, the order would mirror the one used to approach the blue zone of the color chart, as previously discussed. Instead, since the sequence matches the one used to approach the green zone, it can be inferred that the proximity to the light source depends on the volume of radiation remaining in the filtered light.

#### 3.5.2. Approach for the White LED and Cold LED Lamps

The order followed by the transmitted light when using the White LED and Cold LED lamps as illuminants to move closer to the light source in the color chart is reported in Table 8.

It is possible to note that the sequence is not similar to any of those considered. This implies that when the White and Cold LEDs are used as light sources, the relative quantity of radiation with shorter wavelengths contained in the filtered light and the volume of radiation removed from the incident light are not the main factors affecting the distance of the transmitted light from the light source in the color chart.

When discussing Figure 8, we already mentioned that in the case of the White and Cold LEDs, the wavelengths of the greatest part of the radiation contained in the incident light is localized in the blue region of the UV-Vis spectrum. In contrast, considering the other light sources, they are localized in the red region. In that context, the spectral response of the incident light is one of the main factors determining the distance of the filtered light from the light source in the color chart.

**Figure 11:**
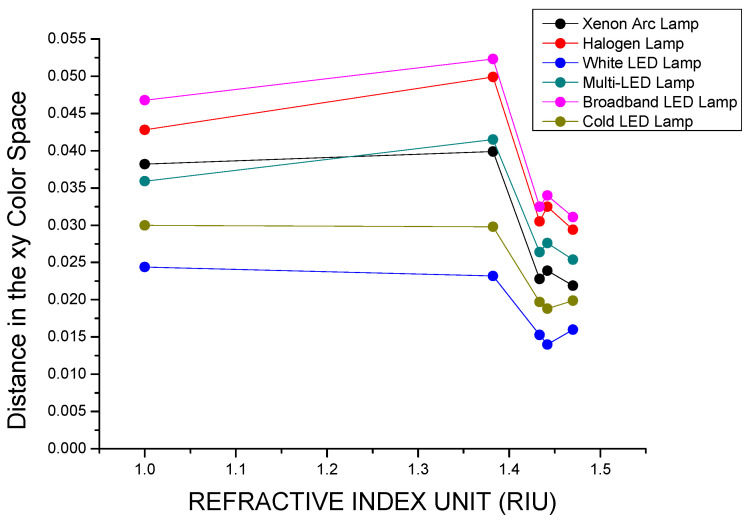
Distances of the light transmitted through the different filters from the light sources calculated as Euclidian distances in the xy color space.

In Figure 11, the color differences, calculated as the Euclidian distance in the *xy* color space between the points representing the light source and the transmitted light, are reported against the values of the refractive index of the medium embedding the plasmonic paper for each one of the illuminants taken into consideration. The Broadband LED lamp displays the largest values for distances, whereas the White LED lamp displays the lowest, and the sequence according to which the distances are reduced for the different illuminants is the following: Broadband LED > Halogen Lamp > Xenon Arc lamp > Multi-LED lamp > Cold LED lamp > White LED lamp.

### 3.6. Variations in the RGB Channels

**Figure 12:**
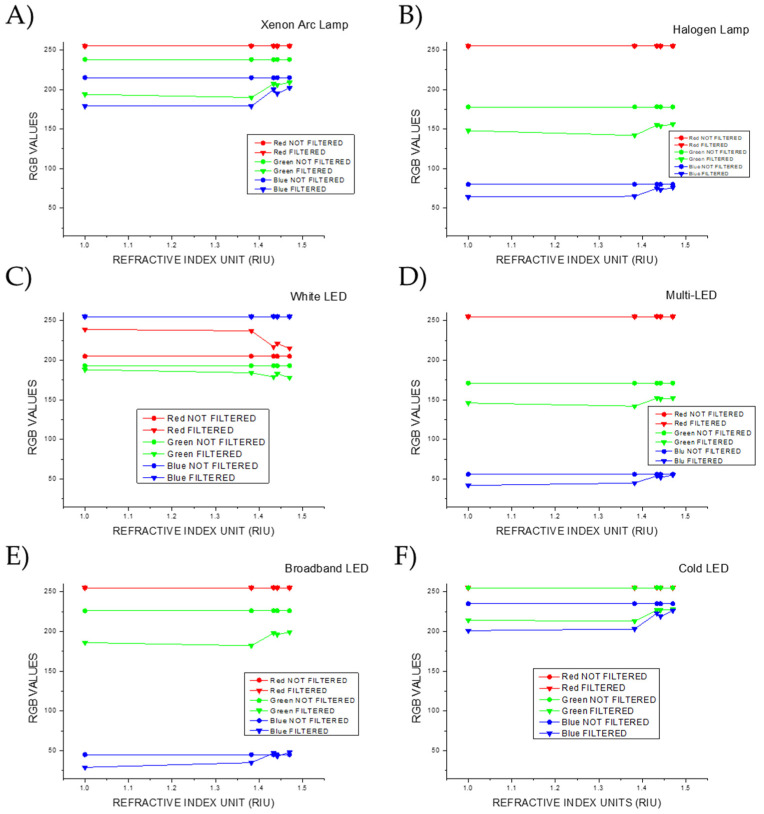
Variations in the single RGB channels for the different RI values of the local environment around the particle surface under illumination of the (**A**) Xenon Arc lamp, (**B**) Halogen lamp, (**C**) LED lamp, (**D**) Multi-LED lamp, (**E**) Broadband lamp, and (**F**) Cold LED lamp.

Figure 12 shows the calculated values for each of the RGB components of the light, both before and after passing through the plasmonic paper, as a function of the refractive index (RI).

Focusing on the red channel, it can be observed that, with the exception of the White LED lamp, the values for both the light source and the transmitted light are at the maximum (255). For the White LED lamp, however, the red channel values of the transmitted light exceed those of the light source, indicating a shift toward the red zone of the color chart when the light passes through the various filters.

Considering the green channel, the transmitted light displays lower values compared to the light source, in line with the radiation removed from the incident light and thus with the filtering properties of the plasmonic paper. When using the Xenon Arc lamp and the Broadband LED, the differences between the values of the light source and the transmitted light for the different RIs are comparable, as can be observed from the graph. Analogous values for the differences are reported for the Cold LED lamp, but the three diverse light sources diverge in the values before crossing the plasmonic paper. The values in the green channel for the light sources decrease in the following order: Cold LED > Xenon Arc Lamp > Broadband LED. The Halogen and Multi-LED lamps display similar values in the differences between the light source and the transmitted light and in the light before crossing the plasmonic paper. The smallest differences in the green channel for the filtered light are observed when using the White LED as light source.

Furthermore, it is possible to note that the order according to which the differences are reduced is the same order in which the transmitted light approaches the green area of the color chart. Here, for the plasmonic paper in solution 2, the point is more distant from green, and for the plasmonic paper in glycerol, the point is closer.

When examining the blue channel, the Xenon Arc lamp and the Cold LED lamp report similar values for the difference between the light source and the transmitted light, with the Cold LED lamp presenting the highest values before crossing the plasmonic paper. The Halogen, Multi-LED, and Broadband LED lamps display comparable values for the differences before and after crossing the filters, and the values of the light sources decrease in the following order: Halogen > Multi > Broadband. The White LED shows the maximum values (255) before and after passing through the plasmonic paper.

As well as for the green channel, observing the color chart in Figure 10, it is possible to note that the order in which the transmitted light approaches the blue zone of the color space is the same order in which the differences decrease, matching the quantity of radiation with shorter wavelengths that is removed from the incident light.

The differences in the colorimetric response of the filtered and not filtered light arising from the filtering properties of the plasmonic paper in the diverse areas of the color chart, and thus also manifested in the separate RGB channels, are the ones detected by the TCS34725 color sensor of the *Color Picker System*.

### 3.7. Real-World Applications

#### 3.7.1. Optimized Light Source to Insert into a Sensing Device

With the aim to transfer the data obtained from the simulation study into real-world applications, it should be considered that a light source with the potential to be integrated into the *Color Picker System* must be able to produce the greatest differences in the output signal for small differences in the detected colors. On this basis, an optimized light source produces the largest distances between two consecutive points on the color chart representing the chromatic response of the filtered light.

**Figure 13:**
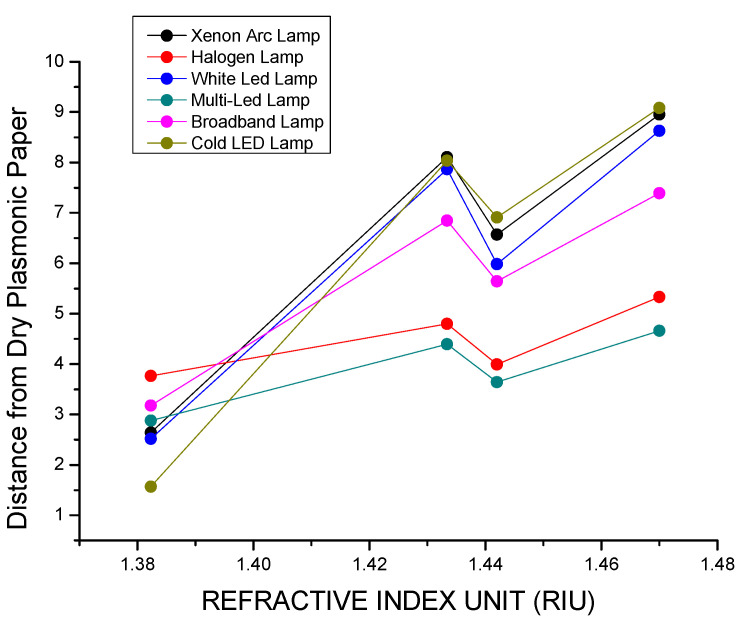
Distances between the light transmitted through the dry plasmonic paper and the other filters, calculated as a color difference based on the CIE76 standard.

In Figure 13, the distances in the color chart between the light transmitted through the dry plasmonic paper and the other filters are reported so that it is possible to evaluate how the transmitted light moves away through the sequence.

The graph shows that the largest distances through the sequence of filters are observed when using the Cold LED lamp as the light source. Similar results are seen with the Xenon Arc lamp, followed by the White LED. In contrast, the changes in distances are less pronounced with the Halogen Lamp and the Multi-LED lamp.

Based on these results, the Cold LED demonstrates the highest potential for integration into the *Color Picker System*. In this case, the light source, in combination with the filtering properties of the plasmonic paper, produces the largest distances in the color chart between consecutive points representing light transmitted through different filters. As a result of these increased distances, the changes in the chromatic response of the filtered light are more pronounced, leading to the highest output signal intensity.

For each light source, the sensitivity toward the chromatic changes in the plasmonic paper is calculated based on the slope of a linear fit to the plot of the distance from the dry plasmonic paper versus the RI (Table 9).

#### 3.7.2. Optimized Light Source in the Separate RGB Channels

Colorimetric analysis differentiates signals obtained from the red, green, and blue channels. This analysis evaluates the differences between the light source and the transmitted light through the sequence of filters for each RGB channel.

**Figure 14:**
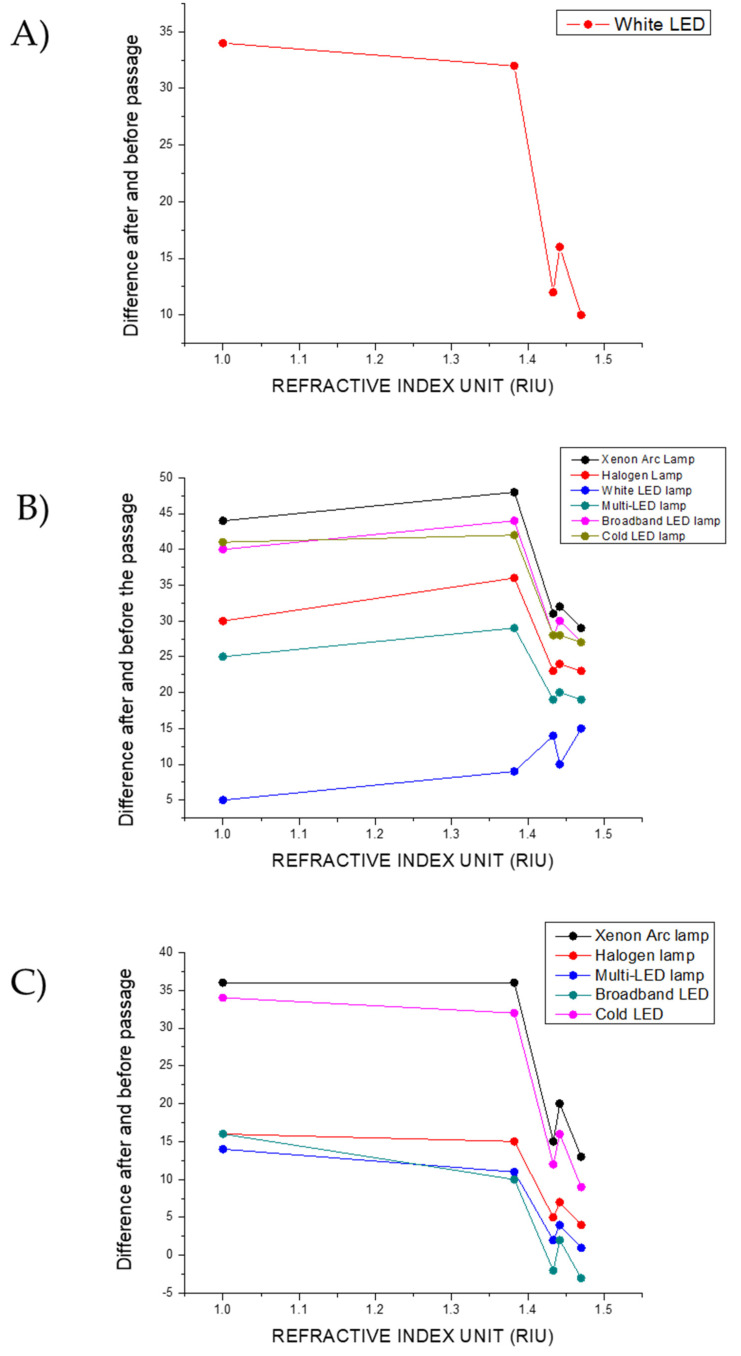
Values for the differences between the transmitted light and the light source in the (**A**) red channel, (**B**) green channel, and (**C**) blue channel for the different filters.

The differences between the transmitted light and the light source for the various plasmonic papers are shown in Figure 14.

Among all the light sources considered, only the White LED lamp showed a difference between the values of the transmitted light and the light source in the red channel (Figure 14A). Therefore, it is the only light source able to produce a signal in devices interrogating that channel.

In the green channel (Figure 14B), the variations in the difference for the various filters are most pronounced in the case of the White LED lamp. The largest values of the differences between the light source and transmitted light are obtained when using other illuminants. Taking into account the different filters, the greatest change is obtained when passing from the plasmonic paper embedded in solution 2 to the plasmonic paper in solution 3, whereas they are notably reduced when considering the other plasmonic papers. This implies that the greatest signal in the green channel is obtained when passing from the plasmonic paper embedded in solution 2 to the plasmonic paper embedded in solution 3, whereas the signal is notably reduced when passing through the sequence from a filter to the successive.

The lowest values in the difference between the light source and the transmitted light are displayed for the White LED, but at the same time, they are more pronounced among the different filters. Therefore, when passing through the sequence from a plasmonic paper to the following, the greatest signal in the green channel is produced. On this basis, the White LED will produce the greatest signal through the sequence of the considered filters along with the best performances in applications requiring evaluations in the green channel.

The Cold LED and the Xenon Arc lamps show the greatest values in the difference between the light source and the transmitted light along with the larger differences through the sequence of the filters in the blue channel (Figure 14C). Therefore, for sensing applications that require measurements in the blue channel, integrating the Cold LED lamp or the Xenon Arc lamp will enhance the system’s performance by providing the greatest signal.

## 4. Conclusions

Herein, the characteristics of nanoparticle arrays incorporated into plasmonic paper, as well as the light source to integrate into the set-up of the Color Picker System to optimize the sensitivity and the precision of the device, have been identified by analyzing the colorimetric response of the light transmitted through the plasmonic paper.

The final filtering properties of the plasmonic paper result from the interaction between transmittance intensity, minimum wavelengths, and the normalized power intensity spectrum of the light source

Scaling up the filtering properties of the plasmonic paper into the design of the device for colorimetric sensing with plasmonic paper, arrays of nanoparticles with high absorbance, inducing a larger difference in the chromatic response of the transmitted light, are more suitable systems to enhance the device’s sensitivity. This also suggests that transmittance in colorimetric measurements of plasmonic paper should be measured at the same point on the plasmonic paper to avoid errors due to variations in transmittance intensity resulting from different particle densities.

By measuring the distance between consecutive points on the color chart, we identified the light source that produces the largest colorimetric signal. Based on the colorimetric response of the transmitted light through the plasmonic paper, the Cold LED lamp emerged as the most suitable light source for integration into the Color Picker System.

When evaluating the RGB channels separately, the White LED was the only source to produce a detectable signal in the red channel, making it a viable option for applications requiring red channel interrogation. Additionally, the White LED produced the most significant variations in the green channel, resulting in the strongest signal. For the blue channel, the Cold LED and Xenon Arc lamps exhibited the most pronounced changes between the light source and the transmitted light, highlighting their potential for implementation in a point-of-care (POC) system.

This study provides new insights into the filtering properties of plasmonic paper and suggests practical considerations for the fabrication of colorimetric sensing devices, such as the individuation of a light source for optimized performances as well as the characteristics of plasmonic nanoparticle arrays at the basis of the analysis.

## Figures and Tables

**Table 1:** Results obtained in the colorimetric sensing of molecules using the TCS34725 color sensor compared with spectroscopic analysis.

Analyte	Limit of Detection (LOD)				Reference
	TCS34725 Color Sensor			Spectrometer	
	Red Channel	Green Channel	Blue Channel		
Iron	0.32 mg L^−1^	0.10 mg L^−1^	0.12 mg L^−1^	0.11 mg L^−1^	[25]
Sunset Yellow		3 μmol L^−1^	3 μmol L^−1^	1 μmol L^−1^	[26]

**Table 2:** Compositions and refractive indices of the ethanol–glycerol mixtures used to change the dielectric environment around the AuNPs surface.

Solvent	$\varphi_{1}$	$\varphi_{2}$	$n_{12}$
Ethanol	1	0	1.36
Mixture 2	0.8	0.2	1.3823
Mixture 3	0.67	0.33	1.4334
Mixture 4	0.5	0.5	1.4420
Glycerol	0	1	1.47

**Table 3:** Values of the (x, y) coordinates for the ITU-R BT.709 primaries in the xy color space.

ITU-R BT.709 Primaries				
	Red	Green	Blue	White Point (D65)
x	0.64	0.30	0.15	0.3127
y	0.33	0.60	0.06	0.3290

**Table 4:** Transmittance intensity and minimum wavelengths reported in decreasing order for the different types of plasmonic paper.

Transmittance Properties of the Plasmonic Paper	
Wavelengths (nm)	Glycerol > solution 4 > solution 3 > solution 2 > dry
Intensity	Glycerol ≅ solution 3 > solution 2 ≅ solution 4 > dry

**Table 5:** Order followed by the transmitted light to move closer to the red, green, and blue areas of the color chart when using light sources different from the White LED.

Zone Of The Color Chart	Order Followed to Approach the Different Areas of the Color Chart
Red	glycerol > solution 3 > solution 4 > dry > solution 2
Green	solution 2 > dry > solution 4 > solution 3 > glycerol
Blue	dry > solution 2 > solution 4 > solution 3 > glycerol

**Table 6:** Sequences according to which the filtered light moves closer to the different areas of the color chart when using the White LED as light source.

Zone of the Color Chart	Order Followed to Approach
Red	glycerol > solution3 > solution 4 > solution 2 > dry
Green	glycerol > solution3 > solution 4 > solution 2 > dry
Blue	dry > solution 2 > solution 4 > solution 3 > glycerol

**Table 7:** Order followed by the transmitted light to move toward the light source when using light sources different from the White and Cold LEDs.

Light Source	Order Followed to Approach the Light Source
Other Illuminants	solution 2 > dry > solution 4 > solution 3 > glycerol

**Table 8:** Sequence according to which the light transmitted through the plasmonic paper approaches the light source when using the White and Cold LEDs as light sources.

Light Source	Order Followed to Approach the Light Source
White LED and Cold LED lamps	dry > solution 2 > glycerol > solution 3 > solution 4

**Table 9:** Colorimetric sensitivity of the plasmonic paper calculated as the slope of a linear fit to the plot in Figure 13 for each different light source.

Light Source	Sensitivity
Xenon Arc Lamp	71.69341
Halogen Lamp	15.8194
White LED	68.58974
Multi-LED	19.38005
Broadband LED	47.46617
Cold LED	87.02322

## Data Availability

Data is contained within the article.
